# The Prevention of Consumption

**Published:** 1905-10-21

**Authors:** J. W. Miller

**Affiliations:** Assistant Medical Officer of Health, Blackburn


### Hospital Clinics.

### THE PREVENTION OF CONSUMPTION.

By J. W. Miller, M.D., D.P.H., Assistant Medical Officer of Health, Blackburn.

Consumption, on account of the heavy mortality which it causes, rightly occupies a prominent position amongst infectious diseases. Fortunately at the present time we are able to take a more hopeful view with regard to its cure owing to the success of sanatorium treatment.

During the last thirty-five years a considerable reduction in the mortality of the disease has taken place—namely, from 2,448 to 1,323 per million—in spite of the fact that the population has increased 50 per cent.

Amongst the chief factors which have produced this desirable reduction one recognises the following: Better housing accommodation, better ventilation in factories and workshops, shorter hours for work, and better sanitation in all directions. Moreover the masses are better educated and are becoming acquainted with the danger of infection in regard to consumption owing to the prominent attention which is being drawn to this disease by sanitary authorities and others.

Prior to the discovery of the bacillus of tuberculosis by Koch in 1882, very little was known regarding the manner in which the disease was disseminated, and his discovery paved the way for more intelligent means for its prevention.

It is now recognised that the way in which infection is produced is through the inhalation or ingestion of the bacilli.

When sputum from a phthisical person becomes dry particles of dust containing the bacilli of consumption are readily inhaled or swallowed. The bacilli may also be swallowed in milk, meat, or other infected food. This latter means is a less potent factor in causing the disease than the former.

The following methods of prevention are being used against the spread of the disease by the latter method: —

1. Improved housing accommodation for cattle and more especially cows.
2. Better lighting, ventilation, and general sanitary conditions of cowsheds.
3. Greater cleanliness as regards cows and also milkers.
4. Elimination of tuberculous cattle from a herd by means of the tuberculin test.

It is more particularly children to whom consumption is conveyed through milk from tuberculous cows, and valuable work on this account is being done by the sanitary authorities of towns such as Liverpool, where the public are supplied at a small cost with sterilised milk. As a precautionary measure all milk should be boiled before use and meat should be thoroughly cooked before it is eaten.

### General Measures of Prevention.

In most of the large towns special attention is being directed to the prevention of spitting by means of notices in public places such as factories and workshops, public buildings, etc., drawing attention to the desirability of avoiding spitting, and, in addition, notices are being posted up in trams and trains.

Although in some towns by-laws are in force very little action is taken when these by-laws are infringed.

One frequently sees expectoration on the floors of railway carriages, in non-smoking as well as smoking; whilst very little is being done to prevent spitting in public-houses. The public-house is now recognised as a dangerous source of infection, and in consequence innkeepers and barmaids and other persons employed in such places suffer a heavy mortality from phthisis.

It would be an improvement if the spittoons, instead of containing sawdust, contained a liquid disinfectant; when sawdust is used drying readily takes place and particles of infection are conveyed about in the air. There is room for better ventilation also and shorter hours for persons employed in such places.

The floors should be washed out daily with a disinfectant solution.

Railway carriages, tramcars, and omnibuses should be disinfected periodically.

### Notification.

In order to deal satisfactorily with the disease, it is important to know of all the cases of consumption present in any town or district and this knowledge can only be obtained by sanitary authorities through notification.

Consumption is not contagious and cannot be dealt with in the same stringent manner as, for example, scarlet fever or enteric fever; if this be recognised there is no difficulty in dealing with the disease, even when notification is compulsory, so long as publicity is not involved.

In New York compulsory notification has been in force during the last ten years, and in 1902 such notification was extended to all forms of tuberculosis.

In New York where the better class consumptives are concerned the physician in attendance is asked to give the particular information desired by the health authority to the patients and their friends and also to inform such authority, if a patient gives up treatment, or removes to another part of the town. A knowledge of the movements of the poorer consumptives is most desirable as they frequently change their residence.

### Bacteriological Examination of the Sputum.

It is most essential that cases should be recognised as early as possible and in many cases great assistance is furnished by a bacteriological examination of the sputum. This work is undertaken free of cost in most of the large towns throughout the country and is largely made use of by medical practitioners. An examination of the sputum should be undertaken in all cases which have been under treatment, as cases apparently cured sometimes contain bacilli in their sputum and are in consequence a source of danger to others.

Special Precautions in the Home.

It is in the house in which consumptives live that the infection is most concentrated, and here there is a danger of persons becoming affected with the disease who are in close contact with persons affected.

Husbands have been known to have been infected by wives and vice versa, whilst it happens sometimes that persons of the same family become affected at the same time or consecutively.

Infection persists for a long time in a house, as the bacilli of consumption retain their vitality for long periods in dark corners away from the light; in the street the bacilli soon lose their vitality owing to free access of light and air.

Similarly the germs retain their vitality in workshops, factories, and public buildings. The most important preventive means are:—

1. *Isolation.*

There can be no question that consumptives should be isolated; in the case of the better class there is no difficulty, but as quite 50 per cent. of all cases occur amongst working-class people isolation is often in the latter case a matter of some difficulty. The majority of houses in which the working class reside contain four rooms, two living rooms, and two bedrooms, and, as the average number of occupants is five, isolation can only take place in one of the living rooms and this cannot be considered satisfactory, as the street door in most cases opens directly into the front room. Removal to sanatoria or other institutions is necessary.

2. *Destruction of Sputum.*

The expectoration may be received on to paper or rags and immediately burnt. A cup or spittoon may be lined with paper, the expectoration can then be lifted up in the paper and burnt and the vessel scalded with boiling water. The expectoration can also be received into a vessel containing disinfectant, a 1 in 20 solution of carbolic acid or a 1 per cent. solution of Izal, and the contents thrown down the drain.

Consumptives when out of doors or whilst at work should use a metal pocket spittoon with a closely fitting lid; it can be lined with paper so that the contents can be burnt and the vessel should afterwards be scalded.

3. *Disinfection.*

Each room in the house should be disinfected every few months.

The walls should first be rubbed over with dough and then washed down with disinfectant. The floors and skirting-board should be washed with a solution of disinfectant weekly.

In some of the larger towns when a death occurs from consumption a notice is sent to the householder offering disinfection by the sanitary authority free of charge, an advantage of which many avail themselves.

It is very important that disinfection should be carried out as removal frequently takes place after a death and in many cases no papering or whitewashing is done before the next occupant takes possession.

Precautions in Regard to Occupation.

Persons affected with the disease are in most cases able to follow their occupation for some months and in some cases for several years; whilst working such persons are a constant source of danger to their fellow-workers; in a good many cases no precautions are taken in regard to spitting and expectoration takes place on to the floor of the workshop, factory, or other room (if indoor) where work is carried on. Where the work is carried on out of doors the affected persons are less dangerous, as the bacilli will be quickly devitalised by free access of light and air.

A time comes when work is no longer able to be performed, and in the case of the working classes this usually means removal to a house at less rent where the rooms are smaller and less food is taken at a time when more nourishment is required. These cases may survive even under such conditions for some months; a good many find their way into the workhouse hospital, others are treated in a general hospital for a time, whilst others drag on at home as best they can.

Consumptives should not be allowed to follow their occupation, but it would be unfair to prohibit employment unless there were institutions for the reception of such persons.

(*To be continued.*)

